# Supplementary material for: Dual-Specificity Phosphatase 1 (DUSP1) Has a Central Role in Redox Homeostasis and Inflammation in the Mouse Cochlea
Source: Antioxidants (Basel). 2021 Aug 25;10(9):1351. doi: 10.3390/antiox10091351 (PMC8467085; doi:10.3390/antiox10091351)
Supplement: Supplementary file 1 [file antioxidants-10-01351-s001.zip › Figure S1-english-changes.pdf]

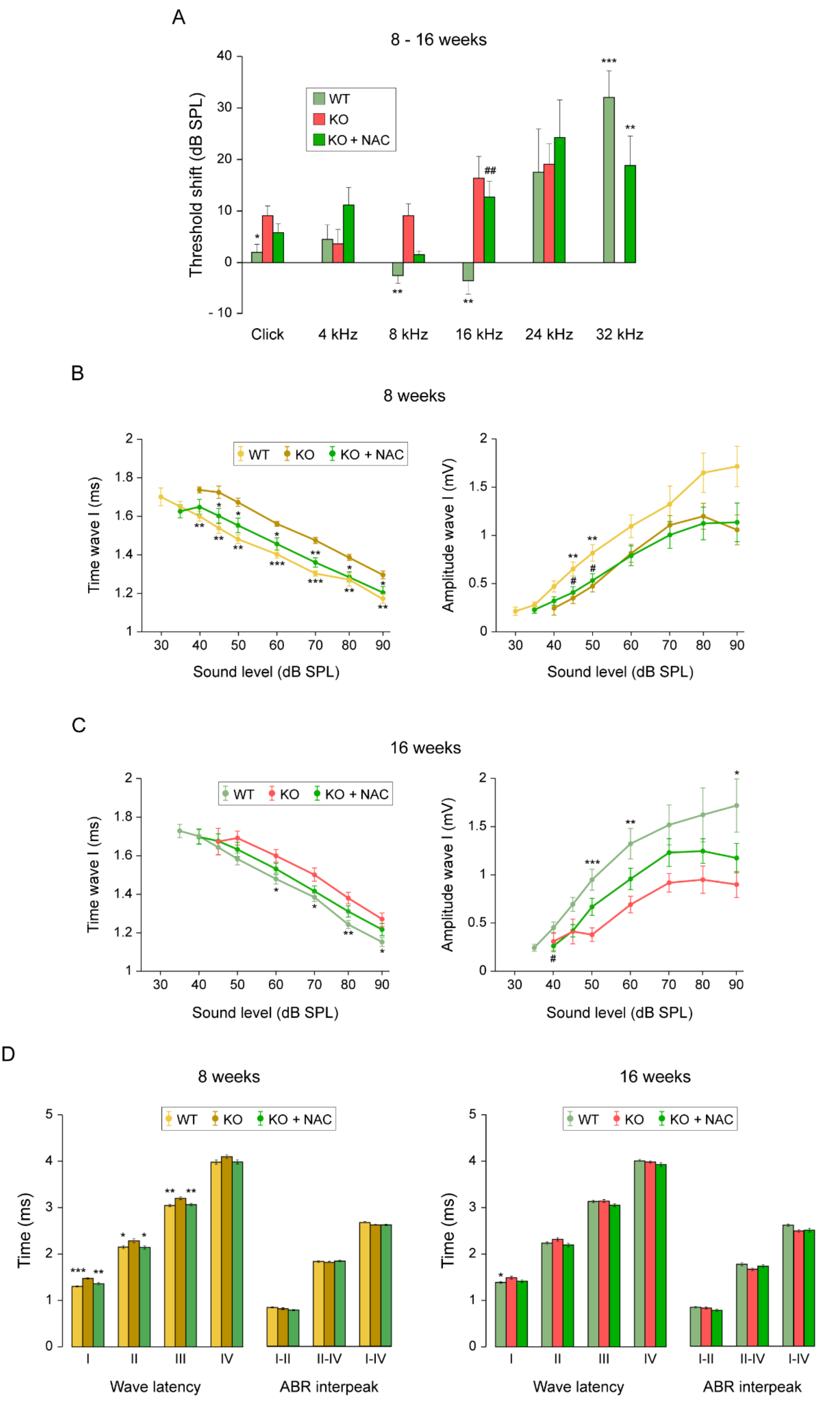

Figure S1. Comparative hearing loss progression and sound conduction efficiency of *Dusp1*$^{+/+}$, *Dusp1*$^{-/-}$ and N-acetylcysteine-treated *Dusp1*$^{-/-}$ mice. (A) Hearing loss progression from 8 to 16 weeks of age shown by auditory brainstem response (ABR) threshold shifts of wild-type (WT), *Dusp1* knock-out (KO) and N-acetylcysteine-treated *Dusp1* KO mice. (B and C) Input-–output ABR wave I latency and amplitude plotted against intensity (dB SPL) for 8- and 16-week-old WT, *Dusp1* KO and NAC-treated *Dusp1* KO mice. (D) ABR waves I, II, III, IV and interpeak latencies (I-II, II-IV and I-IV) comparison at fixed click stimulus intensity (70 dB SPL). All data areis presented as mean ± SEM of at least 15 mice per condition. Statistical significance between genotypes was analyzed by one-way ANOVA: * *vs* KO, # *vs* WT (*, # $p<0.05$; **, ## $p<0.01$; ***, ### $p<0.001$).
