# Supplementary material for: Dual-Specificity Phosphatase 1 (DUSP1) Has a Central Role in Redox Homeostasis and Inflammation in the Mouse Cochlea
Source: Antioxidants (Basel). 2021 Aug 25;10(9):1351. doi: 10.3390/antiox10091351 (PMC8467085; doi:10.3390/antiox10091351)
Supplement: Supplementary file 1 [file antioxidants-10-01351-s001.zip › Tables S1 and S2-edited.pdf]

**Table S1.** Gene sets enriched in *Dusp1*$^{-/-}$ cochlea.			**20-week-old**		**32-week-old**	
**Gene set**	**Database**	**Size**	**NES**	**FDR q-Value**	**NES**	**FDR q-Value**
Immune system	REACTOME	926	6.564	0.00000	4.247	0.00000
Gene expression	REACTOME	760	6.716	0.00000	6.037	0.00000
Metabolism of proteins	REACTOME	590	4.669	0.00000	6.411	0.00000
Cell cycle	REACTOME	479	7.087	0.00000	6.110	0.00000
Pathways in cancer	KEGG	392	2.324	0.00110		
Neuroactive ligand-receptor interaction	KEGG	284	-2.785	0.00010		
DNA repair	REACTOME	262	4.377	0.00000	3.810	0.00000
Organelle biogenesis and maintenance	REACTOME	253			3.191	0.00000
MAPK signaling pathway	KEGG	251	-2.327	0.00200		
Cytokine-cytokine receptor interaction	KEGG	250	3.905	0.00000	2.418	0.00130
Neuronal system	REACTOME	247	-7.933	0.00000	-4.097	0.00000
RAS signaling pathway	KEGG	226	-2.351	0.00180		
Regulation of actin cytoskeleton	KEGG	215			2.781	0.00020
RAP1 signaling pathway	KEGG	214	-2.603	0.00040	2.071	0.00810
Viral carcinogenesis	KEGG	214	3.179	0.00000	2.201	0.00440
Focal adhesion	KEGG	202	2.045	0.00600	2.940	0.00020

cAMP signaling pathway	KEGG	196	-2.358	0.00180	-2.270	0.00890
Chemokine signaling pathway	KEGG	183	3.886	0.00000	2.645	0.00040
Huntington's disease	KEGG	180	4.688	0.00000	2.257	0.00310
Calcium signaling pathway	KEGG	179	-3.159	0.00000		
Purine metabolism	KEGG	176	2.936	0.00000	3.152	0.00000
cGMP-PKG signaling pathway	KEGG	170	-2.266	0.00280		
Phagosome	KEGG	162	2.880	0.00000	3.438	0.00000
Alzheimer's disease	KEGG	162	5.159	0.00000		
JAK-STAT signaling pathway	KEGG	158	2.924	0.00000		
Oxytocin signaling pathway	KEGG	157	-3.948	0.00000		
Hippo signaling pathway	KEGG	153	-3.094	0.00000	-2.453	0.00400
Cell adhesion molecules	KEGG	149	2.187	0.00280	2.646	0.00040
Adrenergic signaling in cardiomyocytes	KEGG	147	-3.484	0.00000		
Non-alcoholic fatty liver disease	KEGG	146	6.713	0.00000		
WNT signaling pathway	KEGG	141	-3.061	0.00000		
Signaling pathways regulating pluripotency of stem cells	KEGG	140	-2.127	0.00580		
Muscle contraction	REACTOME	137	-4.823	0.00000	2.652	0.00010
Serotonergic synapse	KEGG	131	-2.332	0.00200		
Axon guidance	KEGG	128	-4.674	0.00000	-2.694	0.00070
Dopaminergic synapse	KEGG	127	-4.401	0.00000	-2.294	0.00810
AMPK signaling pathway	KEGG	126	2.454	0.00060		

Parkinson's disease	KEGG	126	5.774	0.00000		
Cell cycle	KEGG	123	3.789	0.00000	2.884	0.00020
Platelet activation	KEGG	121	3.605	0.00000	3.371	0.00000
Leukocyte transendothelial migration	KEGG	118	3.996	0.00000	3.949	0.00000
Oxidative phosphorylation	KEGG	118	5.759	0.00000		
Natural killer cell mediated cytotoxicity	KEGG	115	3.672	0.00000		
Glutamatergic synapse	KEGG	113	-5.436	0.00000	-2.787	0.00060
Carbon metabolism	KEGG	113	5.270	0.00000		
Cholinergic synapse	KEGG	111	-4.185	0.00000		
Insulin resistance	KEGG	110	2.675	0.00020		
TNF signaling pathway	KEGG	108	3.510	0.00000		
T-cell receptor signaling pathway	KEGG	105	2.372	0.00080		
DNA replication	REACTOME	104	5.349	0.00000	4.799	0.00000
AGE-RAGE signaling pathway in diabetic complications	KEGG	102	2.669	0.00020		
Pyrimidine metabolism	KEGG	101	3.417	0.00000	2.571	0.00050
Toll-like receptor signaling pathway	KEGG	101	3.150	0.00000		
Retrograde endocannabinoid signaling	KEGG	100	-4.930	0.00000	-3.065	0.00000
Melanogenesis	KEGG	99	-2.907	0.00000		
Estrogen signaling pathway	KEGG	97	-2.134	0.00570		
Circadian entrainment	KEGG	96	-4.086	0.00000		
Phosphatidylinositol signaling system	KEGG	95	-3.154	0.00000	-2.865	0.00040

Chemical carcinogenesis	KEGG	92	2.165	0.00300	2.578	0.00050
NF-κß signaling pathway	KEGG	91	4.271	0.00000		
Morphine addiction	KEGG	90	-4.032	0.00000	-2.716	0.00070
Dilated cardiomyopathy	KEGG	88	-2.407	0.00140		
GnRH signaling pathway	KEGG	88	-2.432	0.00130		
Retinol metabolism	KEGG	88	2.529	0.00030		
GABAergic synapse	KEGG	87	-5.075	0.00000	-2.528	0.00260
Erbß signaling pathway	KEGG	87	-2.020	0.01000	-2.303	0.00800
Programmed cell death	REACTOME	86	2.414	0.00030		
FcγR-mediated phagocytosis	KEGG	86	2.193	0.00280	2.157	0.00510
Insulin secretion	KEGG	85	-3.412	0.00000		
Gap junction	KEGG	85	-2.201	0.00380		
Steroid hormone biosynthesis	KEGG	85	2.700	0.00010		
Aldosterone synthesis and secretion	KEGG	84	-2.398	0.00150		
Hypertrophic cardiomyopathy	KEGG	84	-2.448	0.00120	2.194	0.00450
Small cell lung cancer	KEGG	84	2.593	0.00020		
Peroxisome	KEGG	83	3.884	0.00000		
Apoptosis	KEGG	81	3.074	0.00000		
Salivary secretion	KEGG	78	-2.439	0.00130		
Biosynthesis of amino acids	KEGG	76	2.064	0.00540		
Arrhythmogenic right ventricular cardiomyopathy	KEGG	74	-2.770	0.00010		
Cardiac muscle contraction	KEGG	74	2.007	0.00750		

Gastric acid secretion	KEGG	73	-2.251	0.00300		
Antigen processing and presentation	KEGG	73	2.808	0.00000	2.651	0.00040
Chronic myeloid leukemia	KEGG	73	2.152	0.00320		
B-cell receptor signaling pathway	KEGG	72	3.286	0.00000		
Adipocytokine signaling pathway	KEGG	72	2.975	0.00000		
Viral myocarditis	KEGG	69	2.329	0.00100	2.187	0.00460
Inositol phosphate metabolism	KEGG	69			-2.704	0.00060
RIG-I-like receptor signaling pathway	KEGG	67	2.081	0.00490		
P53 signaling pathway	KEGG	67	2.979	0.00000		
Amphetamine addiction	KEGG	65	-3.566	0.00000		
Renin secretion	KEGG	65	-2.354	0.00180		
Long-term potentiation	KEGG	65	-2.799	0.00000		
Glycolysis / gluconeogenesis	KEGG	63	3.466	0.00000	2.279	0.00280
Cytosolic DNA-sensing pathway	KEGG	62	2.548	0.00020	2.366	0.00160
Autoimmune thyroid disease	KEGG	61	2.364	0.00080		
Synaptic vesicle cycle	KEGG	61	-2.998	0.00000		
Long-term depression	KEGG	60	-2.322	0.00200		
Inflammatory bowel disease	KEGG	58	3.107	0.00000		
Regulation of lipolysis in adipocytes	KEGG	57	2.527	0.00030		
Nod-like receptor signaling pathway	KEGG	57	2.814	0.00000	2.113	0.00630
Acute myeloid leukemia	KEGG	57	1.982	0.00880		
Basal cell carcinoma	KEGG	55	-2.373	0.00170		

Glutathione metabolism	KEGG	55	2.204	0.00260	2.606	0.00050
Type I diabetes mellitus	KEGG	53	2.154	0.00320	2.124	0.00600
Valine. leucine and isoleucine degradation	KEGG	53	3.893	0.00000	-2.387	0.00530
HIV-NEF pathway	BioCarta	52	3.491	0.00000		
Graft-versus-host disease	KEGG	49	3.005	0.00000	2.259	0.00310
Fanconi anemia pathway	KEGG	48	2.487	0.00040	2.077	0.00790
Hedgehog signaling pathway	KEGG	48	-2.213	0.00350		
PPAR-α pathway	BioCarta	48	2.092	0.00860		0.00000
Cocaine addiction	KEGG	47	-2.440	0.00130		
Allograft rejection	KEGG	46	2.148	0.00320	2.090	0.00730
Proteasome	KEGG	45	3.265	0.00000	2.824	0.00010
Keratinocyte pathway	BioCarta	43	2.338	0.00390		
Intestinal immune network for IgA production	KEGG	41	2.669	0.00010	2.167	0.00470
Porphyrin and chlorophyll metabolism	KEGG	41	2.127	0.00360		
Nicotine addiction	KEGG	40	-4.413	0.00000	-2.767	0.00050
Pyruvate metabolism	KEGG	39	3.990	0.00000		
Pentose and glucuronate interconversions	KEGG	36	2.153	0.00320		
DNA replication	KEGG	35	4.448	0.00000	3.954	0.00000
IL-2Rb pathway	BioCarta	34	2.592	0.00160		0.00000
GPCR pathway	BioCarta	33	-2.626	0.00070		
Base excision repair	KEGG	33	2.436	0.00060		
Citrate cycle	KEGG	32	4.461	0.00000	-2.372	0.00540

Toll pathway	BioCarta	31	2.199	0.00450		
IL-1R pathway	BioCarta	31	2.319	0.00370		
Propanoate metabolism	KEGG	31	4.596	0.00000		
Death pathway	BioCarta	30	2.695	0.00110		
Pentose phosphate pathway	KEGG	30	2.857	0.00000		
RNA polymerase	KEGG	29	1.981	0.00880		
Glyoxylate and dicarboxylate metabolism	KEGG	29	3.029	0.00000		
TNFR1 pathway	BioCarta	28	2.232	0.00400		
Homologous recombination	KEGG	28	3.309	0.00000		
Ascorbate and Aldarate metabolism	KEGG	27	1.980	0.00880		
NKT pathway	BioCarta	27	2.532	0.00180		
FAS pathway	BioCarta	26	2.235	0.00410		
Stress pathway	BioCarta	25	2.720	0.00080		
Asthma	KEGG	23	2.504	0.00040	2.203	0.00450
PGC1-α pathway	BioCarta	23	-2.512	0.00090		0.00040
Mismatch repair	KEGG	22	2.264	0.00160	2.381	0.00140
Cell cycle pathway	BioCarta	22	2.225	0.00390		
NF-κB pathway	BioCarta	22	2.578	0.00150		
RACCYCD pathway	BioCarta	22	2.154	0.00580		
G2 pathway	BioCarta	21	2.323	0.00390		
NOS1 pathway	BioCarta	20	-3.399	0.00000		
Proteasome pathway	BioCarta	20	2.308	0.00360	2.659	0.00750

Caspase pathway	BioCarta	20	3.614	0.00000	
ATR BRCA pathway	BioCarta	20	2.416	0.00350	
IL-12 pathway	BioCarta	20	2.369	0.00390	
Mitochondria pathway	BioCarta	19	2.853	0.00030	
Chemical pathway	BioCarta	19	2.300	0.00330	
2-oxocarboxylic acid metabolism	KEGG	19	2.283	0.00140	
TNFR2 pathway	BioCarta	18	2.090	0.00830	
NK cells pathway	BioCarta	18	2.175	0.00500	
ATM pathway	BioCarta	17	2.291	0.00340	
RELA pathway	BioCarta	16	2.099	0.00850	
P53 pathway	BioCarta	15	2.278	0.00350	
NO2IL-12 pathway	BioCarta	15	2.882	0.00040	

Normalized enrichment value (NES); False discovery rate (FDR).

Table S2. TaqMan probes and primers for RT-qPCR experiments.

Symbol	Gene	Reference
Nlrp3	NLR family Pyrin Domain Containing 3	Mm00840904_m1
Kim1	Kidney injury molecule 1	Mm00506686_m1
Il18	Interleukin 18	Mm00446190_m1
Il10	Interleukin 10	Mm00446145_m1

Gene	Forward Primer (5'-3')	Reverse Primer (5'-3')
Keap1	ACAGCAGCGTGGAGAGATATGA	CCAATCCTCCGTGTCAACATT
Nqo1	GGTAGCGGCTCCATGTACTC	CATCCTTCCAGGATCTGCAT
Sod1	CCAGTGCAGGACCTCATTTT	TTGTTTCTCATGGACCACCA
Sesn2	CGTGGCTCATCACCAAGGA	CAGCTGTGCTCGCCAGTCT
Cs	AGGCTACACCGACCCTCAGTT	ATGGTCACTATGGATGGTGAGGTA

Primers were designed using Primer Express 3.0 software and the mouse gene sequences available on the Ensembl genome database.
